# RETRACTION: Evaluating the Role of Wound‐Healing Genes in Conjunction With Stool Routine and Serum Tumor Markers for Colorectal Cancer Diagnosis and Prognostic Implications

**DOI:** 10.1111/iwj.70540

**Published:** 2025-04-18

## Abstract

**RETRACTION:** YuK. and FangX. , “Evaluating the Role of Wound‐Healing Genes in Conjunction With Stool Routine and Serum Tumor Markers for Colorectal Cancer Diagnosis and Prognostic Implications,” International Wound Journal 21, no. 3 (2024): e14768, 10.1111/iwj.14768.38446012 PMC10916745

The above article, published online on 06 March 2024, in Wiley Online Library (http://onlinelibrary.wiley.com/), has been retracted by agreement between the journal Editor in Chief, Professor Keith Harding; and John Wiley & Sons Ltd. Following an investigation by the publisher, all parties have concluded that this article was accepted solely on the basis of a compromised peer review process. In addition, the investigation found that the authors provided incomplete ethical approval information for the study. The editors have therefore decided to retract the article. The authors did not respond to our notice regarding the retraction.

**RETRACTION:** K. Yu and X. Fang , “Evaluating the Role of Wound‐Healing Genes in Conjunction With Stool Routine and Serum Tumor Markers for Colorectal Cancer Diagnosis and Prognostic Implications,” International Wound Journal 21, no. 3 (2024): e14768, 10.1111/iwj.14768.38446012 PMC10916745

The above article, published online on 06 March 2024, in Wiley Online Library (http://onlinelibrary.wiley.com/), has been retracted by agreement between the journal Editor in Chief, Professor Keith Harding; and John Wiley & Sons Ltd. Following an investigation by the publisher, all parties have concluded that this article was accepted solely on the basis of a compromised peer review process. In addition, the investigation found that the authors provided incomplete ethical approval information for the study. The editors have therefore decided to retract the article. The authors did not respond to our notice regarding the retraction.

